# Rituximab and Bendamustine (BR) Compared with Rituximab, Bendamustine, and Cytarabine (R-BAC) in Previously Untreated Elderly Patients with Mantle Cell Lymphoma

**DOI:** 10.3390/cancers13236089

**Published:** 2021-12-03

**Authors:** Giulia Bega, Jacopo Olivieri, Marcello Riva, Greta Scapinello, Rossella Paolini, Silvia Finotto, Roberto Sartori, Elisa Lucchini, Gianmarco Guandalini, Davide Facchinelli, Maria Chiara Tisi, Marco Basso, Laura Ballotta, Francesco Piazza, Isacco Ferrarini, Carlo Visco

**Affiliations:** 1 Department of Medicine, Section of Hematology, University of Verona, 37129 Verona, Italy; giulia.bega@aulss9.veneto.it (G.B.); gianmarco.guandalini18@gmail.com (G.G.); isacco.ferrarini@univr.it (I.F.); 2 Hematology and SCT Unit, Azienda Sanitaria Universitaria Integrata Santa Maria della Misericordia, 33100 Udine, Italy; jacopo.olivieri@asufc.sanita.fvg.it; 3 Cell Therapy and Hematology, San Bortolo Hospital, 36100 Vicenza, Italy; marcello.riva@aulss8.veneto.it (M.R.); davide.facchinelli@aulss8.veneto.it (D.F.); mchiara.tisi@gmail.com (M.C.T.); 4 Department of Medicine, Section of Hematology, University of Padova, 35122 Padova, Italy; greta.scapinello@gmail.com (G.S.); francesco.piazza@unipd.it (F.P.); 5 Oncohematology, Santa Maria della Misericordia Hospital, 45100 Rovigo, Italy; rossella.paolini@aulss5.veneto.it; 6 Oncology 1 Unit, Department of Oncology, Istituto Oncologico Veneto IOV-IRCSS, 35128 Padova, Italy; silvia.finotto@iov.veneto.it; 7 Onco Hematology Unit, Istituto Oncologico Veneto IOV-IRCSS, 31033 Castelfranco Veneto, Italy; roberto.sartori@iov.veneto.it (R.S.); marco.basso@iov.veneto.it (M.B.); 8 Hematology Unit, Azienda Sanitaria Universitaria Giuliano-Isontina, 34148 Trieste, Italy; elisa.lucchini@asugi.sanita.fvg.it (E.L.); laura.ballotta@asugi.sanita.fvg.it (L.B.)

**Keywords:** mantle cell lymphoma, bendamustine, R-BAC, elderly, therapy

## Abstract

**Simple Summary:**

Both BR, and R-BAC are suitable induction therapies in elderly patients with mantle cell lymphoma (MCL). However, the two regimens have not been compared before. We retrospectively analysed the outcome and the safety features of elderly patients with newly diagnosed MCL, treated with BR or R-BAC between 2008 and 2019 at eight institutions. We used propensity scores to reduce selection bias, thus analysing 156 patients (53 BR, 103 R-BAC). Patients treated with R-BAC achieved higher CR rate than BR (91% vs. 60%, *p* < 0.0001). The 2-year PFS was 87 ± 3% and 64 ± 7% for R-BAC and BR, respectively (*p* = 0.001). Median overall survival (OS) was 121 months for R-BAC and 78 months for BR (*p* = 0.08). R-BAC was associated with significantly more pronounced grade 3–4 thrombocytopenia than BR (50% vs. 17%). This study indicates that R-BAC is associated with significantly prolonged 2-year PFS than BR in elderly patients with MCL.

**Abstract:**

Background: Rituximab plus bendamustine (BR), and rituximab, bendamustine, and cytarabine (R-BAC) are well-known induction therapies in elderly patients with mantle cell lymphoma (MCL), according to clinical guidelines. However, a direct comparison between the two regimens has never been performed. Methods: In this multicentre retrospective study, we compared the outcome of patients with newly diagnosed MCL, treated with BR or R-BAC. Primary endpoint was 2-year progression-free survival (PFS). Inclusion bias was assessed using a propensity score stratified by gender, age, MCL morphology, and MIPI score. Results: After adjusting by propensity score, we identified 156 patients (53 BR, 103 R-BAC) with median age of 72 (53–90). Median follow-up was 46 months (range 12–133). R-BAC was administered in a 2-day schedule or with attenuated dose in 51% of patients. Patients treated with R-BAC achieved CR in 91% of cases, as compared with 60% for BR (*p* < 0.0001). The 2-year PFS was 87 ± 3% and 64 ± 7% for R-BAC and BR, respectively (*p* = 0.001). In terms of toxicity, R-BAC was associated with significantly more pronounced grade 3–4 thrombocytopenia than BR (50% vs. 17%). Conclusions: This study indicates that R-BAC, even when administered with judiciously attenuated doses, is associated with significantly prolonged 2-year PFS than BR in elderly patients with previously untreated MCL.

## 1. Introduction

Mantle cell lymphoma (MCL) is an aggressive form of non-Hodgkin lymphoma characterized by continuous relapses over time, with no standard initial therapy for patients who are not eligible for an autologous transplant [1]. Furthermore, many patients cannot be eligible for intensive therapies because of their older age, or medical comorbidities. [2] The standard-of-care upfront therapy for elderly or frail patients was represented by R-CHOP (rituximab, cyclophosphamide, doxorubicin, vincristine, and prednisone), followed by maintenance with rituximab [3] or by the VR-CAP regimen, where vincristine is replaced by bortezomib [4]. Alternatively, in the last decades, bendamustine-based therapies have been increasingly adopted worldwide for elderly patients with MCL. Two phase 3 randomized studies have investigated the role of the bendamustine and rituximab (BR) in this setting as opposite to R-CHOP. The first study reported that BR had fewer toxicities and significantly improved progression-free survival (PFS) in comparison with R-CHOP [5]; the second study showed that BR had better long-term disease control than R-CHOP/R-CVP, confirming that BR represents a suitable first-line option for patients with MCL [6]. With the aim of improving the efficacy of the BR regimen, the R-BAC regimen (rituximab, bendamustine, and intermediate dose cytarabine) has been proposed by the Fondazione Italiana Linfomi (FIL) as a very active alternative induction regimen for elderly patients with MCL [7]. When indirectly comparing historical results, with the limitations of such analysis, it appears that BR was less toxic than R-BAC (especially on blood counts), but also less active. The STiL [5], and FIL study [7] had apparently similar populations, with median age of 70 (64.5–74) and 71 (67–75), respectively, but in the first patients treated with BR had a median PFS of 35.4 months, as opposite to 76% 3-year PFS of R-BAC [7]. Similarly, in the Bright study [6], median PFS after long-term follow up for the BR arm approximated 48 months [8].

European and international clinical guidelines [9,10] list the two bendamustine-based regimens as feasible options in MCL patients who are not eligible for autologous transplant. However, to the best of our knowledge no study so far has compared face-to-face the efficacy of these two regimens.

As in the Veneto region both BR and R-BAC represent standard induction treatment for elderly patients with MCL, we performed a retrospective survey with the aim of comparing the two regimens in similar populations of patients from our geographical area.

## 2. Materials and Methods

### 2.1. Study Design and Partecipants

This was a multicentre, observational, retrospective study that enrolled patients from eight centres of the Rete Ematologica Veneta (REV). The two regimens (BR and R-BAC) represented routine induction regimens in the selected centres for patients with MCL not eligible for autologous transplant. Due to the retrospective nature of the study, treatment assignment to one therapy instead of the other was based on local doctor decision. Patients were included if they: (i) were previously untreated; (ii) were not eligible for upfront autologous transplant; (iii) had established histological diagnosis of MCL, made by an expert pathologist according to the criteria of the WHO classification [11]. Reasons for not being eligible for upfront autologous transplant in patients less than 65 years-old were represented by coexisting comorbidities or medical conditions, since all enrolling institutions routinely treat with BR or R-BAC transplant ineligible patients.

Clinical and pathological data of each included patient were retrieved by local investigator from medical records, after obtaining written informed consent. The study was performed in accordance with the principles of the Declaration of Helsinki and Good Clinical Practice. The study was denominated “BE-ve-BAC study”, and was approved by the Ethics Committee of Verona University on 24 March 2021, protocol number 18448.

### 2.2. Efficacy and Toxicity Outcomes

The primary efficacy outcome was 2-year PFS defined as progression, relapse or death from any cause two years after diagnosis. Secondary outcomes were overall survival (OS), defined as the time from diagnosis until death from any cause, response to treatment (either overall response and complete remission rate), and toxicity. OS from time of first relapse (OS-2) was defined as the time from first relapse to death for any cause. Tumour response was assessed at the end of induction treatment irrespective of the number of administered cycles. For response assessment, since not all included patients were staged with PET at the end of therapy, we adopted Cheson 2008 criteria [12]. Toxicity was measured by means of number of patients that interrupted treatment prematurely for reasons not related to tumour response, or by registered episodes of relevant toxicity, defined as grade 3–4 haematological toxicity, grade 3–4 nonhaematological toxicity.

### 2.3. Statistical Analysis

Demographics and clinical patient characteristics were summarized using descriptive statistical methods. All patients treated with at least one cycle of either BR or R-BAC were included, provided they had a minimum follow-up of 12 months since start of treatment. Inclusion bias was addressed by using a propensity score (PS), that was calculated based on possible known confounding factors, which were gender, age, MCL blastoid morphology, and MIPI score. In order to reduce selection bias, we applied an inverse probability of treatment weight (IPTW). The PS was estimated using a multivariate logistic model, with the type of treatment (BR versus R-BAC) that represented the dependent variable and the covariates listed above were the possible confounding factors.

The survival analysis was estimated using the Kaplan–Meier method and the groups were compared using log-rank test. Multivariate analysis was performed using Cox regression models. Toxicity, completed treatment rates and treatment response rates were compared with the chi-square or Fisher’s test.

## 3. Results

### 3.1. Patients

Overall, 180 patients with MCL with a median age of 72 years (range 53–90) were identified and included in the database. According to our IPTW calculation, the probability of receiving R-BAC instead of BR was significantly higher in younger patients (*p* < 0.0001), with no additional significant difference in the distribution of other computed prognostic variables (*p* = 0.31 for gender, *p* = 0.73 for morphology, *p* = 0.15 for MIPI score). Therefore, we performed our comparative analysis in patients who were 80 years old or younger, in order to smooth this significant age difference between the two cohorts, and to obtain a fair comparison between the two groups. This final cohort included 156 patients (53 BR, 103 R-BAC), which represented the subject of the present analysis. The clinical and pathological characteristics of these patients, then divided for treatment allocation, are shown in Table 1. Of them, 109 (70%) were males, MIPI was elevated in 64.1%, 11.5% had blastoid or pleomorphic morphology, 47% had high Ki 67 index. As reported in Table 1, the BR and R-BAC group appeared comparable in terms of disease characteristics and main prognostic factors at presentation, except for age, as previously discussed.

### 3.2. Administered Cycles, Dose Reductions, and Tumor Response

Overall, 146 patients (94%) received at least four induction cycles. Of the 10 patients who were treated with less than four cycles, eight were treated with BR (15%), and two with R-BAC (2%, *p* = 0.002). The main reasons for not administering at least four cycles were tumour progression (seven patients: six BR, one R-BAC) or toxicity (three patients: two BR, one R-BAC).

Cycles were reduced in total dose in 62 patients. Approximately half (51%) of patients treated with R-BAC had substantial dose reductions, which were mainly represented by the 2-day schedule (instead of 3-day), meaning that the third day of cytarabine was skipped. The detailed distribution of administered cycles and dose reductions are reported in Table 2. A similar proportion of patients in the two study groups (12% for BR, 14% for R-BAC) delayed the interval between cycles of more than 2 weeks.

Complete response (CR) was achieved in 126 patients (81%); 12 patients (8%) were primary refractory to induction therapy. According to Cheson 2008 criteria, patients treated with R-BAC achieved CR in 76% of cases, as compared to 47% of patients treated with BR (*p* = 0.0004). When we analysed patients staged by PET-scan (N = 80), according to Lugano criteria, patients treated with R-BAC achieved CR in 91% of cases, as compared to 60% of patients treated with BR (*p* < 0.0001). Accordingly, only three patients treated with R-BAC had refractory disease (3%), as opposed to nine patients treated with BR (17%, *p* = 0.001).

### 3.3. Survival Analysis

**Figure 1:**
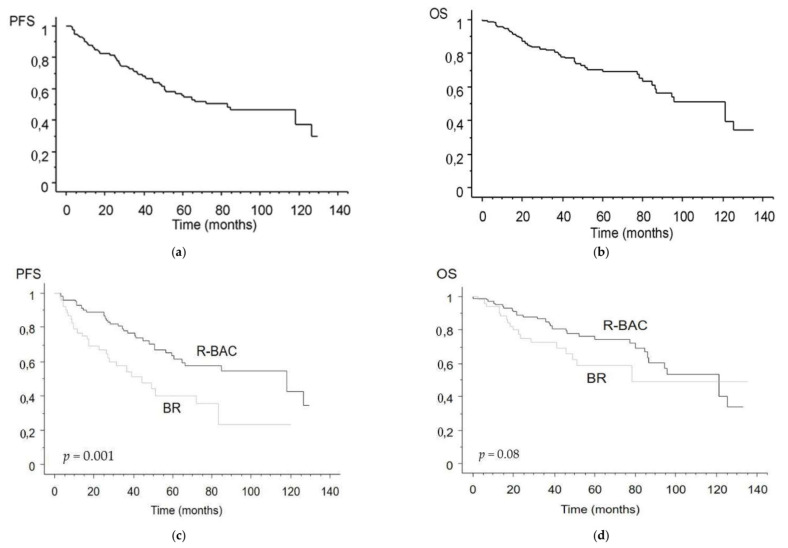
PFS (**a**) and OS (**b**) of all 156 MCL patients. PFS (**c**) and OS (**d**) of all 156 MCL patients divided according to administered upfront treatment.

Overall, 49 patients died during the study period. Of them, 32 (65%) died of progressive disease, four (8%) of second neoplasms (three prostatic carcinoma and one sarcoma), two of cardiac complications, and 11 of a miscellanea of other causes. None of the patients died of infection. Median follow-up for survivors from MCL diagnosis was 46 months (range 12–135), with no statistical difference between BR (42 months, range 12–135) and R-BAC (52 months, range 12–133) cohorts (*p* = 0.12 by Mann–Whitney test). The 2-year PFS was 87% ± 3% and 64% ± 7% for R-BAC and BR, respectively (*p* = 0.001). Median overall survival (OS) was 121 months for R-BAC and 78 months for BR (*p* = 0.08, Figure 1).

For the whole series of 156 patients, high MIPI score was the only predictive significant variable both in terms of PFS and OS (*p* = 0.001 and *p* = 0.003, respectively). The same high MIPI score was predictive of adverse PFS (but not OS) in patients treated with R-BAC, while it was associated with significantly inferior PFS and OS in patients treated with BR.

We analysed survival of the 127 patients > 65 years (50 treated with BR, 77 with R-BAC) matched across the treatment programs, and we found that R-BAC was associated with significantly superior PFS (*p* = 0.01), but not OS (*p* = 0.386).

Patients treated with R-BAC who had dose reductions had similar outcome than those who received full dose.

### 3.4. Toxicity

As mentioned above, three patients interrupted prematurely the induction therapy due to toxicity. Reasons were prolonged neutropenia grade 4 (one patient), and erythematous skin reaction grade 4 (two patients).

As shown in Table 2, we registered 208 episodes of relevant toxicity, which were evenly distributed among the two treatment groups. Patients receiving R-BAC experienced 133 episodes of grade 3–4 haematological toxicity: 32 grade 3–4 anaemia, 49 grade 3–4 neutropenia and 52 grade 3–4 thrombocytopenia. After R-BAC, we registered eight cases of febrile neutropenia as compared to two in the BR group (*p* = NS). Other causes of nonhaematological toxicity in the R-BAC group were infections (four patients), skin reactions (five patients), or miscellaneous other causes in the remaining 15 patients.

Of note, in the BR group we recorded significantly less cases of severe thrombocytopenia (*p* = 0.004).

### 3.5. Second Line Treatments and Survival, POD-24

Of the 64 patients who experienced first-relapse during the study period, 10 patients did not receive second line therapy, and were managed with supportive therapy or palliative care due to rapid tumour progression or old age and poor performance status.

**Figure 2:**
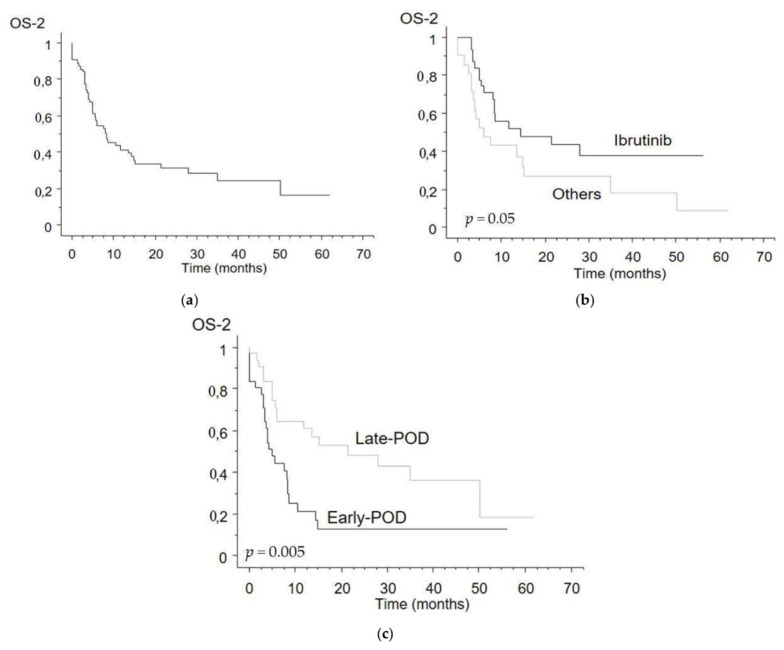
Overall survival from time of first relapse (OS-2) of MCL patients: (**a**) all 64 relapsed or refractory patients; (**b**) patients who had ibrutinib as second line (*n* = 33) versus other treated patients (*n* = 21); (**c**) patients experiencing early- (*n* = 31) versus late-POD (*n* = 33) after first line.

Thirty-three of the 54 treated patients (61%) were treated in second line with ibrutinib. Remaining patients were treated with anthracycline-based therapy (7), bortezomib-based regimens (4), lenalidomide (2), bendamustine-based (5), and other different chemotherapeutic regimens (3). Overall, OS-2 was 8.2 months (Figure 2a). The median OS-2 for patients treated with ibrutinib was significantly longer than for patients treated with other approaches (15.2 months versus 6.0 months, *p* = 0.05, Figure 2b).

Furthermore, patients that were refractory to induction therapy or who experienced early progression of disease (POD), defined as POD within 24 months from start of therapy (POD-24, *n* = 31), had significantly inferior median OS-2 than patients with late-POD (5 months versus 21 months, *p* = 0.005, Figure 2c). When divided according to first line, patients with POD-24 were more represented among the BR patients (21 of 28 POD, 75%), than among the R-BAC patients (10 of 36 POD, 28%, *p* = 0.0002). The detrimental effect of POD-24 on patient final outcome was observed both in the BR and in the R-BAC treated patients.

## 4. Discussion

With the present report, we show that R-BAC was more effective than BR in an unselected series of consecutive elderly patients with MCL from our geographical area. This difference was particularly pronounced in terms of PFS, while curves showed only a trend in favor of R-BAC in terms of OS. This improvement in efficacy was at the cost of increased haematotoxicity, specifically thrombocytopenia.

Available comparison of historical results had suggested that R-BAC was associated with superior survival, but with increased haematotoxicity than BR. However, to our knowledge, the present study represents the first attempt of face-to-face comparison between BR and R-BAC, since the two regimens had never been compared before, either retrospectively or prospectively. Our results may assist physicians’ choice when allocating patients to a bendamustine-based approach, as opposite to R-CHOP-based induction therapy. According to our observations, R-BAC may then be considered the best choice in terms of efficacy, but be destined cautiously to relatively younger or fit patient populations. Since toxicity of R-BAC was mainly limited to blood counts, and was transient, we believe that judicious dose reductions may be applied to the R-BAC regimens in less fit or elderly patients, facilitating a wider use of this regimen. Indeed, the 2-day schedule was widely adopted in the everyday practice described in this trial, and did not hamper efficacy results, while sparing toxicity, as previously reported [7]. It is not surprising that most of the included patients received R-BAC and not BR, since R-BAC has been conceived in this territory, and well used by most centers in or outside trials of the FIL (Fondazione Italiana Linfomi).

We acknowledge that the major limitation of our study was represented by the retrospective collection of patients. Propensity score matching and other statistical methods cannot incorporate unmeasurable confounders such as physiological age which is a major factor in making treatment decisions in astute clinicians. However, the concern for a selection bias may have been attenuated by the enrollment of consecutive patients from centers where the two regimens were routinely used as standard induction. Moreover, it is likely that the use of propensity score further smoothed the selection bias, although we cannot exclude the contribution of unknown confounders.

The fact that the survival advantage of R-BAC was limited to PFS was not surprising since the median age of our patients was 72 years, and only 65% of our patients died of progressive disease, meaning that in many cases the cause of death had little to do with the type of first line regimen.

We have shown that ibrutinib represents a valid second-line option for patients relapsing after bendamustine-based induction. The survival expectancy of relapsing patients was significantly improved by the availability of ibrutinib as a second line option. Ibrutinib monotherapy had similar efficacy after BR or R-BAC (*p* = 0.25 for OS-2). Overall, our real-life data show a relatively inferior OS-2 than other series of patients enrolled in clinical trials [13,14].

In parallel to what has been described in younger patients [15,16], POD24 confirmed its high discriminative power in elderly patients, as shown in Figure 2c; as observed by others [17], and mirroring results of younger cohorts [15], ibrutinib was the best choice in early-POD (*p* = 0.006 in favor of ibrutinib versus other choices), but not in late-POD (*p* = 0.42).

With the CAR-T cell therapy era at the door, the use of bendamustine-based regimens before T-cell lymphocyte harvest has been debated, due to the detrimental impact on lymphocyte health and functions. Therefore, this drug may be dedicated to elderly population, especially above 70 years, due to its excellent activity and overall toxicity profile. Furthermore, since median PFS with both regimens exceeded 3 years, most patients will benefit from a sufficient time interval to allow T-lymphocytes to recover their initial function.

## 5. Conclusions

The BE vs. BAC study indicates that R-BAC, even when administered in the 2-day schedule or with attenuated dose, was associated with significantly more prolonged PFS than BR in elderly patients with previously untreated MCL. As hypothesized hematological toxicity was significantly higher for R-BAC regimen.

Long term follow-up of the R-BAC500 prospective study (NCT01662050, reference number 7) of the Fondazione Italiana Linfomi (FIL) is awaited to confirm our findings in an independent prospective setting.

## Figures and Tables

**Table 1:** Baseline characteristics.

Variation	Data (*n* = 156)	BR (*n* = 53)	R-BAC (*n* = 103)	*p*-Values
Median age (years)	72 (53–80)	73 (53–80)	69 (54–80)	0.012
Sex				
Male	109 (70%)	37 (70%)	72 (70%)	0.890
Female	47 (30%)	16 (30%)	31 (30%)	
Ann Arbor stage				
I–II	11 (10%)	4 (8%)	7 (7%)	0.862
III–IV	145 (90%)	49 (92%)	96 (93%)	
Bone marrow involvement				
Yes	127 (81%)	52 (98%)	75 (73%)	0.623
No	29 (19%)	1 (2%)	28 (27%)	
Morphological variants				
Classical	138 (89%)	46 (87%)	92 (89%)	0.901
Pleomorphic-Blastoid	18 (11%)	7 (13%)	11 (11%)	
Ki 67 index				
<30%	73 (47%)	21 (40%)	52 (50%)	0.442
≥30%	47 (30%)	14 (26%)	33 (32%)	
n.d.	36 (23%)	18 (34%)	18 (18%)	
MIPI				
Low risk	11 (7%)	5 (10%)	6 (6%)	0.751 *
Intermediate risk	45 (29%)	15 (28%)	30 (29%)	
High risk	100 (64%)	33 (62%)	67 (65%)	

Data are *n* (%) unless indicated otherwise. MIPI = Mantle Cell Limphoma International Prognostic Index. * calculated as MIPI high vs. others.

**Table 2:** Administered cycles, premature interruptions, and toxicity.

Variation	BR (*n* = 53)	R-BAC (*n* = 103)	*p*-Value
Number of cycles			
<4	8 (15%)	2 (2%)	0.001
≥4	11 (21%)	41 (40%)	0.016
6	34 (64%)	60 (58%)	0.474
Dose reduction			
≥25%	10 (19%)	52 (51%)	0.008
Two-day schedule		42 (41%)	
Toxicities			
Anaemia grade 3–4	8 (15%)	32 (31%)	0.121
Neutropenia grade 3–4	20 (38%)	49 (48%)	0.531
Thrombocytopenia grade 3–4	9 (17%)	52 (50%)	0.004
Nonhaematological toxicity grade 3–4	13 (24%)	25 (24%)	1.000

## Data Availability

The data presented in this study are available on request from the corresponding author. The data are not publicly available due to privacy.
